# The Oncomodulatory Role of Human Cytomegalovirus in Colorectal Cancer: Implications for Clinical Trials

**DOI:** 10.3389/fonc.2014.00314

**Published:** 2014-11-17

**Authors:** Hsin-Pai Chen, Yu-Jiun Chan

**Affiliations:** ^1^Department of Medicine, National Yang-Ming University Hospital, Yilan, Taiwan; ^2^School of Medicine, National Yang-Ming University, Taipei, Taiwan; ^3^Division of Infectious Diseases, Department of Medicine, Taipei Veterans General Hospital, Taipei, Taiwan; ^4^Institute of Public Health, School of Medicine, National Yang-Ming University, Taipei, Taiwan; ^5^Division of Microbiology, Department of Pathology and Laboratory Medicine, Taipei Veterans General Hospital, Taipei, Taiwan

**Keywords:** human cytomegalovirus, colorectal cancer, oncomodulation, anti-cancer immunity, anti-cancer therapy

## Abstract

Increasing evidence suggests that human cytomegalovirus (HCMV), a beta-herpes virus that chronically infects human beings, is associated with colorectal cancer (CRC). The viral nucleic acids specifically localized to the neoplastic mucosal epithelium of CRC, while tumoral presence of HCMV independently predicted a poor outcome in elderly patients. In the past decade, the concept of “oncomodulation” of HCMV in human cancers has been formulated. In CRC, changes in the tumor microenvironment are closely related to cancer behavior and prognosis, while the underlying mechanism driving these changes remains unclear. As HCMV affects multiple cellular functions, including signal pathways that regulate angiogenesis, apoptosis, cell invasiveness, and anti-cancer immunity, the virus potentially exerts oncomodulatory effects in the tumor microenvironment of CRC. Here, we summarize the current knowledge about the association between HCMV and CRC and suggest future perspectives on both research and anti-cancer therapy of CRC.

## Introduction

Colorectal cancer (CRC) is one of the most commonly found cancers leading to significant mortality and morbidity. Despite efforts and progress made in the treatment, the survival rate is still low unless found and treated early (1). Multiple factors contribute to the outcome of CRC. The gold standard for outcome prediction is based on the tumor–node–metastasis classification of the Union for International Cancer Control (TNM-UICC). However, since the pathogenesis of CRC involves multiple and heterogeneous pathways, an observation based merely on anatomy and histology may sometimes be misleading. Patients of the same stage frequently have dramatically different outcomes. Such dis-concordance may result from changes in tumor microenvironments that have been shown to be closely associated with prognosis (2). Nevertheless, the underlying mechanisms driving these microenvironmental changes in cancer behavior and subsequent local anti-cancer immunity in the tumor microenvironment remain obscure.

Virus as an oncogenic agent has long been recognized, for example, human hepatitis B and C viruses to hepatoma and human papilloma virus to cervical cancer. A certain herpes group virus, such as Epstein-Barr (EB) virus, has been shown to relate to nasopharyngeal carcinoma and Burkitt’s lymphoma. However, human cytomegalovirus (HCMV), a beta-herpesvirus that contains many potential viral onco-proteins, has not been categorized as an oncogenic virus till now. This virus infects 60–90% of the general population and is regarded as an oncomodulatory virus because of its effects on cell-cycle progression, mutagenesis, angiogenesis, and immune evasion (3–5). It was a long debate whether HCMV may act as an oncomodulatory virus. First, whether HCMV can be found in various tumorous tissues is skeptical, although its gene components and gene products had been detected in a number of human cancers (6–10). It is much clearer now HCMV can be detected in a certain tumors, including CRC. In CRC, several studies have identified HCMV in the tumor tissues (11–13). We previously reported that ∼40% of the CRC specimens were positive for HCMV (13). Later, we found tumoral presence of HCMV is associated with an unfavorable outcome in elderly CRC patients and predicts a shorter disease-free survival, independent of that expected by the TNM stage (14). Such findings imply that HCMV may affect the tumor microenvironment of CRC via a certain immune pathway. Theoretically, upon infection, HCMV is able to up-regulate different host cellular signal pathways, growth factors, and cytokines, resulting in enhanced cell survival, proliferation, and angiogenesis (15). As such, HCMV appears to regulate the malignant behavior of tumor cells, implying an oncomodulatory role for the virus.

## The Association between HCMV and Outcome of CRC: Increasing Evidence

### HCMV preferentially infects the tumoral epithelium of colorectal cancer

Using paired tumor and adjacent non-neoplastic CRC specimens, we recently reported that HCMV preferentially infects the neoplastic epithelium of CRC. HCMV DNA was detected by PCR in 42.3% (69/163) of the tumor specimens, while only 5.6% (14/163) samples of adjacent non-neoplastic tissue were positive for HCMV (*p* < 0.0001) (13). Significantly higher viral copies were found in the tumor specimens than the adjacent non-neoplastic tissue specimens. By *in situ* hybridization, the nucleic acids of HCMV specifically localized to the cytoplasm of neoplastic epithelium, but not the submucosa, stroma, or inflammatory infiltrations surrounding the tumor epithelium. These results indicate that HCMV preferentially infects, or reactivates in, the tumor epithelium of CRC, and is concordant with several previous studies that reported positive detection of HCMV in CRC (11, 12).

However, detection of HCMV in CRC had been associated with controversial results. (16–19). Negative detection of HCMV may be attributed to several factors. Among the various techniques used, a PCR-based detection of viral nucleic acids is both rapid and sensitive, and is the most widely applied on detecting HCMV in clinical samples. However, the selection of genes, the primer design, and the quality and type of samples can all have substantial influence on the result (20, 21). PCR detection of viral nucleic acids may fail if the tissue has been processed extensively by formalin, which causes fragmentation of DNA (22, 23). Frozen samples, on the other hand, are more suitable for DNA preservation and subsequent PCR detection (24, 25). Moreover, HCMV may only infect malignant cells that are at specific stages of cell differentiation and maintains a “low-grade infection” status in tumor (20, 26, 27). If the sample volume is too small, a false negative detection is expected.

### Tumoral presence of human cytomegalovirus is associated with shorter disease-free survival in elderly patients with CRC

The relationship between HCMV and the outcome of CRC was demonstrated in our recent study in which an association between HCMV and a poor survival outcome in the elderly patients was found. Significant interaction is seen between HCMV and the elderly. In the elderly, HCMV tends to get reactivated more frequently because of the age-related decline of cell-mediated immunity (28, 29). On the other hand, chronic cytomegaloviral infection is detrimental to the older population, associated with increased mortality in elderly (30). The underlying mechanism may be that chronic HCMV infection results in phenotypic and functional alterations of adaptive immunity in the elderly, a status referred to as “immunosenescence” (31–34).

We recently investigated the relationship between tumoral presence of HCMV and the outcome of elderly patients with CRC. In the 81 patients who underwent curative surgery, the 39 patients with HCMV-positive tumors had a lower disease-free survival rate compared with those without HCMV in tumor (*P* = 0.024). For patients with stage II or III diseases, tumoral HCMV status correlated with disease-free survival more closely than the tumor-node-metastasis (TNM) staging method. Using a multivariate Cox proportional-hazards model, tumoral infection by HCMV independently predicted disease recurrence in 5 years (hazard ratio, 4.42; 95% confidence interval, 1.54–12.69; *P* = 0.006). It is surprising that within specific tumor stages, tumoral presence of HCMV correlated with the outcome more closely than the traditional histopathological staging method. Such finding argues for an important role that the virus may play in the tumor microenvironment of CRC.

## Perspectives of the Oncomodulatory Role of HCMV in CRC

### HCMV and anti-cancer immunity in the cancer microenvironment

#### HCMV and tumor-associated macrophages

Tumor-associated macrophages (TAMs) are derived from blood monocytes that are recruited to the tumor. Macrophages can be activated to either M1, the anti-tumor, or M2, the pro-tumor, polarization states depending on the microenvironment stimuli. The M1 macrophages are part of the T-helper (T_H_) 1 response and are potent effectors against intracellular pathogens and tumor cells. On the other hand, the M2 macrophages are involved in promoting angiogenesis, tissue remodeling and repair, and are part of the T_H_2 response. TAMs with M2 polarization are a major tumor-infiltrating cell population (35). High TAM density in tumors is now recognized as a poor prognostic sign in CRC (36).

When directly infecting the monocyte, HCMV renders monocyte differentiation toward an M1 macrophage (37). However, according to a recent study of human glioma, HCMV encodes an interleukin (IL)-10 homolog, the cmvIL-10, which binds to the human IL-10 receptor. Binding of cmvIL-10 renders the monocytes in glioma to assume an M2 phenotype and turns the anti-cancer immunity toward the immunosuppressive and tumor-propagating phenotype (38). It would be valuable to study whether the presence of HCMV in CRC was also associated with a tumor-propagating phenotype of TAM.

#### HCMV and tumor-infiltrating T cells

Using immunohistochemical staining, quantitative PCR, and large-scale flowcytometry, recent studies have demonstrated the critical role of localized adaptive immune reaction in the prognosis of cancer behavior and prognosis (39, 40). The T cell-mediated immunity within the tumor tissue predicts the outcome of CRC independently to that of the traditional TNM-UICC stage of the tumor (40). In CRC, a shift from T_H_1 to T_H_2 response in CD4^+^ lymphocytes was noted (41). Such shift from T_H_1 to T_H_2 polarization of CD4^+^ lymphocytes renders the tumor-specific cytotoxic CD8^+^ inactive. CRC also shows high levels of MHC class-I alteration, with loss or downregulation of class-I MHC molecules resulting from alteration in processing or presentation (42, 43). The overall effect is immunosuppression and tolerance of tumor growth. The underlying mechanism driving the alteration in T cell-mediated immunity within the tumor microenvironment remains obscure.

Interestingly, HCMV infection results in an immunosuppressive status resembling to that found in CRC. To escape the surveillance of cytotoxic T cells, HCMV encodes several viral genes (*US*2, 3, 6, 11) that downregulate the level of MHC class-I molecules on the surface of infected cells (44). In organ transplant recipients, who suffer from active HCMV diseases, the transcription of IL-10 and transforming growth factor (TGF)-β is significantly increased, while the expression of interferon (IFN)-γ is reduced, suggesting a shift from pro-inflammatory T_H_1 to the immune-tolerogenic T_H_2 response and resemble that found in CRC (45, 46).

In a small number of CRC samples, we analyzed the transcription levels of signal pathways of the major T_H_ subsets, including T_H_1, T_H_2, regulatory T (T_reg_), and a newly defined subset, the T_H_17 that has recently been correlated to a poorer outcome of CRC (47, 48). We found a higher transcription level of IL 17 – the signature cytokine of T_H_17 cells – in HCMV-positive tumors (14). Based on this finding, we speculate that HCMV may be one of the underlying forces that drive the alteration of anti-tumor immunity in the tumor microenvironment of CRC.

### HCMV infection and the cellular signal pathways that correlate with cancer progression

#### HCMV and cyclooxygenase-2 pathway

Cyclooxygenase (COX) is a key enzyme in the prostaglandin biosynthetic pathway. COX-2, the inducible isoform of COX, has received considerable attention due to its role in human cancers. Activation of the COX-2 activity in tumor tissue leads to promotion of angiogenesis, resistance to apoptosis, immune modulation, and increase of cell invasiveness and metastasis (49–51). In CRC, overexpression of COX-2 in CRC tissues correlates with poor prognosis (52), while inhibition of the COX activity by non-steroidal anti-inflammatory drugs (NSAIDs) results in protective effects for CRC (53–55).

Human cytomegalovirus is also an inducer of COX-2, which plays an important role in viral replication (56). In permissive cells, inhibition of COX-2 results in marked reduction of HCMV (57). It was previously reported that COX-2 expression in CRC was concordant with areas of HCMV IE1-72 and pp65 immunoreactivity (12).

#### HCMV may promote angiogenesis in cancer

Induction of angiogenesis is one of the key factors that promote tumor growth and cancer survival. The microvascular density of a tumor has prognostic significance and predicts survival in patients with CRC (58).

Mounting evidence suggests that HMCV may promote angiogenesis. HCMV-infected cells directly induce angiogenesis by secreting VEGF and other angiogenic factors (59), promoting vascular tube formation (60). The HCMV gene *US*28 was shown to be involved in the HCMV-induced angiogenic phenotype (61). Analysis of clinical glioblastoma specimens revealed that *US*28 co-localized with several factors of angiogenesis (62).

#### HCMV promotes cell proliferation and inhibits apoptosis

Human cytomegalovirus is found to promote cell proliferation in cancer. In a mouse model of glioblastoma, cytomegalovirus infection enabled the malignant cells to survive and multiply, leading to shorter survival (63). HCMV also encodes multiple antiapoptotic proteins, including the viral mitochondrion-localized inhibitor of apoptosis (vMIA) or *UL*37 exon 1, *UL*36, and *UL*38 proteins, to prevent host cells from programed death and to support viral survival (64, 65).

#### HCMV infection alters the expression of matrix metalloproteinases that are important in cancer metastasis

Matrix metalloproteinases (MMPs) are zinc-dependent endopeptidases. These enzymes are capable of degrading extracellular matrix proteins and play a major role on cell behaviors such as cell proliferation, migration, differentiation, angiogenesis, apoptosis, and host defense (66). The expression levels of some MMPs are correlated with stage of disease and/or prognosis (67).

Human cytomegalovirus infection is associated with a significant alteration of MMPs in mRNA, protein, and functional levels (68). Among them, MMP-9 negativity is associated with poor survival, while its positivity predicts a favorable outcome of CRC (69). A reduction in MMP-9 mRNA, protein, and activity levels but increased tissue inhibitor of matrix proteinases (TIMP)-1 mRNA and protein levels was found after HCMV infection (68). MMP-9 mRNA expression was affected by an immediate-early or early viral gene product, whereas TIMP-1 mRNA expression was affected by late viral gene products. Furthermore, a higher mRNA level of MMP-1, a marker for hematogenous metastasis of CRC (70), had been demonstrated in HCMV-positive clinical specimens (71).

### Can anti-HCMV treatment change the outcome of CRC?

Traditional treatments for CRC include surgery, radiotherapy, and chemotherapy. Introducing an anti-viral agent or anti-viral immunotherapy for CRC has never been tested. Based on aforementioned evidence for HCMV as an oncomodulatory agent, it is justifiable to test the hypothesis that treating elderly patients whose tumor is positive for HCMV may improve the outcome.

For HCMV infection, there are currently four anti-viral drugs licensed for treatment: ganciclovir, valganciclovir, cidofovir, and foscarnet (Table 1). Ganciclovir is a synthetic analog of deoxy-guanosine. It is the first anti-viral agent approved for treatment of cytomegaloviral disease and remains the first-line treatment (72). Valganciclovir is the L-valyl ester prodrug of ganciclovir, with excellent bioavailability equivalent to that of intravenous ganciclovir. Both drugs have the major adverse effect of cytopenia. Cidofovir is a broad-spectrum anti-viral agent with potency against many DNA viruses including smallpox virus (73). However, severe renal toxicity limits its use as an anti-viral agent. Foscarnet is usually considered as second-line therapy for patients who are failing ganciclovir therapy due to major side effects or viral resistance (72).

**Table 1:** **Anti-viral agents for human cytomegalovirus**.

Drug name	Pharmaceutical preparation	Major adverse effects
Ganciclovir	Mainly intravenous	Cytopenia, teratogenicity/carcinogenicity and aspermia in animals
Valganciclovir	Oral	Same as ganciclovir
Cidofovir	Intravenous	Nephrotoxicity
Foscarnet	Intravenous	Nephrotoxicity

Supportive data from glioblastoma trial may render this notion promising. In a randomized double-blind trial, patients with HCMV-positive glioblastoma were treated with valganciclovir as an add-on therapy. The results demonstrated beneficiary prognosis for this subset of patients (74). By the same token, a subset (15–40%) of HCMV-positive CRC patients may gain benefits from anti-viral treatment. A prospective randomized controlled trial is critical to confirm the hypothesis.

## Conclusion

Increasing clinical and experimental findings suggest that HCMV may be an oncomodulatory virus for human CRC. However, the definite role of HCMV needs to be further investigated in a systematic manner to obtain a comprehensive and holistic understanding. From bench to bed, the clinical relevance of the oncomodulating effects should be testified through a multifaceted approach. Moreover, a randomized controlled trial is needed to clarify whether anti-viral therapy is beneficial to CRC patients with HCMV detected in the tumor. The results of such studies may provide a new insight into the pathogenesis of at least a subset of CRC and hopefully bring about new strategies for cancer therapy.

## Conflict of Interest Statement

The authors declare that the research was conducted in the absence of any commercial or financial relationships that could be construed as a potential conflict of interest.
